# Therapeutic Indications of the Salts of Silver

**Published:** 1898-03

**Authors:** 


### THERAPEUTIC INDICATIONS OF THE SALTS OF SILVER.

Dr. Thos. L. Coley, of Philadelphia, in the *Medical and Surgical Reporter*, concludes an excellent paper on the Therapeutic Indications of the Salts of Silver, as follows:

1. Locally upon all inflammation of mucous membranes, especially acute inflammations where an antiseptic, anti-corrosive, superficial caustic is needed. In leg ulcers where the inflammatory reaction is marked—*i. e.*, not in *sluggish* ulcers. In laryngitis, pharyngitis, faucitis, tonsilitis, etc.
2. Locally painted over furuncles, felons, etc.
3. In the summer diarrheas of infants by high injections.
4. In dysentery, mucous colitis, enteritis and gastro-enteritis, and typhoid fever in adults combined with internal administration of the drug.
5. In the stomach, in ulcer, erosion, dilatation, acid dyspepsia, nervous gastritis, etc.
6. In the nervous system, in tabes dorsalis.

Internally administered the nitrate is best given before meals, combined with opium or simply gum tragacanth.—*The Medical Fortnightly.*

